# Flexible Magnetic Metasurface with Defect Cavity for Wireless Power Transfer System

**DOI:** 10.3390/ma15196583

**Published:** 2022-09-22

**Authors:** Le Thi Hong Hiep, Bui Xuan Khuyen, Bui Son Tung, Quang Minh Ngo, Vu Dinh Lam, Thanh Son Pham

**Affiliations:** 1 Institute of Materials Science, Vietnam Academy of Science and Technology, 18 Hoang Quoc Viet, Cau Giay, Hanoi 100000, Vietnam; 2 Graduate University of Science and Technology, Vietnam Academy of Science and Technology, 18 Hoang Quoc Viet, Cau Giay, Hanoi 100000, Vietnam; 3 Fundamental of Fire Engineering Faculty, University of Fire Prevention and Fighting, 243 Khuat Duy Tien, Thanh Xuan, Hanoi 100000, Vietnam; 4 University of Science and Technology of Hanoi, Vietnam Academy of Science and Technology, 18 Hoang Quoc Viet, Cau Giay, Hanoi 100000, Vietnam

**Keywords:** magnetic metamaterial, magnetic resonance wireless power transfer, coupling resonance, metasurface

## Abstract

In this paper, we present a flexible magnetic metamaterial structure for enhancing the efficiency of wireless power transfer (WPT) systems operating at 13.56 MHz. The metasurface between transmitter (Tx) and receiver (Rx) coils of the WPT system is constructed of a 3 × 5 metamaterial unit cell array with a total size of 150 × 300 mm^2^. Most metamaterial structures integrated into WPT systems are in planar configurations with a rigid substrate, which limits practical applications. The proposed metasurface is fabricated on an FR-4 substrate with a thin thickness of 0.2 mm; therefore, it can be bent with radii greater than 80 mm. A defect cavity is formed in the non-homogeneous metasurface by controlling the resonant frequency of the unit cell with an external capacitor. Simulation and measurement results show that the efficiency of the WPT system is significantly enhanced with metasurfaces. The performance of the WPT system can also be optimized with suitable bend profiles of metasurfaces. This proposed flexible metasurface could be widely applied to WPT systems, especially asymmetric, bendable, or wearable WPT systems.

## 1. Introduction

In recent years, wireless power transfer (WPT) has developed rapidly and grown into a field with many critical applications in industry and commerce [1]. WPT is an advanced technology that allows energy to be transmitted without connecting wires. Along with the advancement of science and technology, mobile devices such as smartphones, smartwatches and other wearable devices have become an indispensable part of human life [2]. WPT technology supports the design of electrical systems to become lean, aesthetically pleasing and convenient. Electric vehicles have also been booming in recent years, reducing greenhouse gas emissions so that the world moves toward a green industry. Wireless charging for these vehicles is also a solution to help them be completed and user-friendly [3,4,5]. In addition, WPT has great potential for applications in implantable devices, space satellites, autonomous underwater vehicles (AUVs), and so on [6,7,8,9].

As early as the early 20th century, Nikola Tesla was experimentally investigating contactless power transmission systems [10]. Over time, this technology has developed and has been used in military and medical applications. C. Brown conducted a far-field WPT experiment at 2.45 GHz in 1963 [11]. Energy is transmitted through electromagnetic waves at long distances, but the efficiency is very low due to significant losses in the transmission line. Near-field WPT, based on the fundamental principle of mutual induction between two coils, has been an active research direction for medical implants since the 1970s [12]. The inductive power transfer (IPT) system for wireless charging of mobile devices was extensively studied in the 1990s and 2000s [13,14]. In 2007, the research group at the Massachusetts Institute of Technology (MIT) led by Prof. Marin Soljacic renewed WPT technology with the idea of magnetic resonance WPT (MR-WPT). This WPT system can light a 60 W bulb at a distance of two meters with an efficiency of 40%. However, the size of the resonant coils is quite large, with a diameter of 60 cm [15].

Recently, the MR-WPT technology has allowed power to be transmitted further than IPT. However, it still has an inherent weakness because the transmission efficiency is very sensitive to the distance between transmitter and receiver resonators. The system performance degrades rapidly as the transmission distance increases. In 2011, Wang et al. proposed the use of metamaterials to overcome this weakness [16]. Metamaterials are artificial structures with unique properties that respond to electromagnetic waves, something which is impossible in natural materials. They were proposed theoretically by Veselago in 1967 and first experimentally built in 2000 by Smith [17,18]. Metamaterials and metadevices can be applied in various new technology fields, such as active microwave camouflage, antennas, absorbers, acoustics, and biosensors [19,20,21,22,23,24,25,26]. Studies on the use of magnetic metamaterials (MMs) to enhance WPT efficiency have been intensively carried out over the past decade and continue to be active now [27,28]. MM can be used to improve WPT systems’ performance in 2D and 3D profiles, as studied by Ranaweera et al. [29]. The metasurface used in WPT planar applications for smart tables has been suggested by Song et al. [30]. In order to increase the applicability of WPT systems, a reconfigurable metamaterial structure was proposed by Shan et al. [31]. In addition, zero-permeability metamaterials can also be used to block magnetic fields to increase safety, as per a study by Lu et al. [32]. Various configurations of metamaterials have been integrated into WPT systems, such as superconducting metamaterials, coding metasurfaces, side-place metamaterials, and wearable metasurfaces [33,34,35,36,37]. Most metamaterial structures integrated into WPT systems are in planar configurations with a rigid substrate, which limits practical applications. Therefore, a flexible metamaterial structure to increase the integration capacity, as well as the application range of the WPT system, is a necessity.

In this paper, we propose a flexible magnetic metasurface with a defect cavity to enhance the efficiency of MR-WPT systems. The effect of a metasurface on WPT efficiency is investigated in both simulation and measurement. The WPT efficiency can be increased or decreased in the bent state depending on the bending radius. While the WPT system still maintains high performance with a radius greater than 80 mm. The optimum transfer efficiency of 55.1% is achieved at a 150 mm bending radius of the non-homogeneous metasurface, compared to the 11.5%-efficiency of the original MR-WPT system.

## 2. Design and Analysis of Flexible Magnetic Metasurface for Wireless Power Transfer

**Figure 1:**
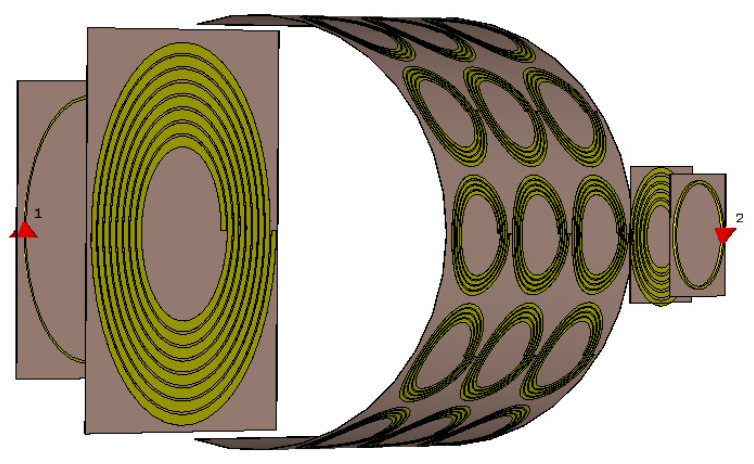
Schematic of the WPT system with a flexible metasurface.

Figure 1 shows the configuration of the proposed WPT system integrated with a bendable metasurface. This is an asymmetric WPT system with the transmitter resonator being three times larger than the receiver resonator. The transmitter part consists of a transmitter resonator (Tx) with an outer diameter of *D*_Tx_ = 150 mm, and the source loop has a diameter of *D*_S_ = 100 mm. In the receiver part, the outer diameter of the receiver resonator (Rx) is *D*_Rx_ = 50 mm, and the diameter of the load loop is *D*_L_ = 40 mm. A bendable metasurface is inserted in between Tx and Rx resonators. This metasurface consists of 3 × 5 unit cells and has a total size is 150 × 300 mm^2^. Each end of the metasurface in the longitudinal direction has 25 mm extra for practice bending. A cavity can be formed by controlling the resonant frequency of unit cells in the metasurface. The cavity formation condition is that the cavity’s resonant frequency must be higher than that of other unit cells. The mechanism of the cavity can be explained by the Fano interference effect between unit cells. Constructive interference occurs when the resonant frequency of the cavity is higher than that of other unit cells so that the magnetic field can be localized in the cavity region. If this is not the case, then destructive interference occurs, leading to degradation of the magnetic field. A 10% difference in frequency is optimal for field confinement [38]. The appearance of the cavity breaks the uniformity of the metasurface. Therefore, a homogeneous metasurface turns into a non-homogeneous metasurface. Our approach aims to improve the performance of the WPT system through homogeneous and non-homogeneous metasurfaces [27]. In this case, the metasurface can be bent up to a minimum radius of 80 mm, thereby increasing the applicability of WPT systems.

**Figure 2:**
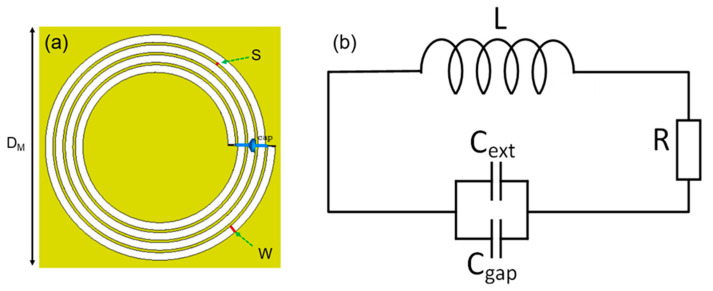
(**a**) Design of MM unit cell, (**b**) equivalent electrical model of MM unit cell.

The methods of designing magnetic metamaterials are diverse. In particular, the *π*-shape spiral is a unique geometry used to fabricate MM in various spectral regions [27]. For magnetic metamaterials that operate in the MHz frequency region, the original π-shape structure needs to be combined with an external capacitor to lower the structure’s natural resonant frequency. Figure 2a depicts a schematic of an MM unit cell designed by a 4-turn spiral resonator loaded with a lumped capacitor. The spiral is fabricated on a thin FR-4 substrate with a thickness of 0.2 mm and a dielectric constant of 4.3. The thickness of the copper strip is 0.07 mm. The spiral has a strip width *W* = 1.5 mm, an inter-strip spacing *S* = 0.5 mm, and an outer radius of 46 mm. The MM unit cell has a square shape with a total size of *D*_M_ = 50 mm. Figure 2b shows a simplified MM unit cell electrical model. The internal parameters of the MM unit cell, such as the series resistance, self-inductance, and self-capacitance, are expressed by *R*, *L* and *C*_gap_, respectively. An external capacitor is added to adjust the resonant frequency of the MM unit cell represented by *C*_ext_.

The resonant frequency of an MM unit cell load with an external capacitor can be calculated as [39]:

(1) $$f_{0}=\frac{1}{2\pi \sqrt{L\left(C_{gap}+C_{\mathrm{ex}t}\right)}}$$

**Figure 3:**
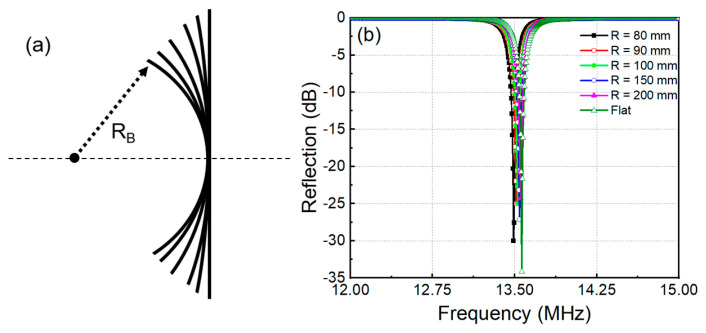
(**a**) Schematic of bending metasurface, (**b**) the resonant frequency of MM unit cell following bending radius.

The bendable metasurface is applied to enhance the transfer efficiency and increase the applicability of the WPT system. Therefore, the response of metamaterial to the bending is a property that needs to be investigated. Figure 3a depicts a schematic of bending metasurfaces with various bending radii denoted by *R*_B_ from 80 to 200 mm, and a flat configuration with a bending radius is infinity. The reflection responses of MM unit cells with different bending radii are shown in Figure 3b. At the flat configuration, the MM unit cell is designed to resonate at the frequency of 13.56 MHz. When the metamaterial is bent in a circular arc with radii from 200 mm to 80 mm, the resonant frequencies slightly change from 13.55 to 13.5 MHz. Because of interactions between the unit cells, a resonant frequency band is formed instead of the resonant peak in the metasurface spectrum [28]. Therefore, a slight change in the resonant frequency of the MM unit cell when subjected to bending does not significantly affect the performance of the metasurface.

**Figure 4:**
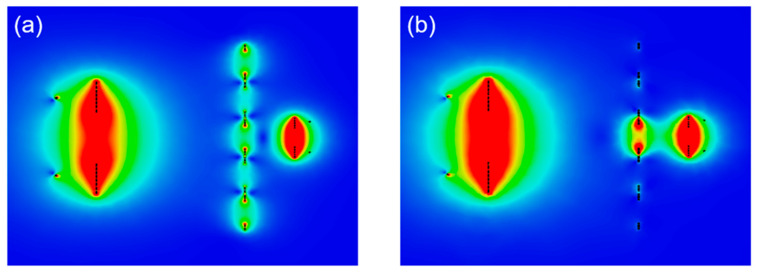
Field distributions in WPT systems (**a**) with a homogeneous metasurface and (**b**) with a non-homogeneous metasurface.

Electromagnetic (EM) simulations were performed by CST Studio Suite software to investigate the field distribution of the WPT system at the resonant frequency of 13.56 MHz. The simulations were implemented with appropriate open boundary conditions applied in all directions. Two discrete ports were used to excite the source loop and load loop. Figure 4a shows the H-field intensity distribution of the WPT system with a homogeneous metasurface. The surface waves appear on the metasurface owing to magneto-inductive waves propagation. Therefore, the metasurface can increase the H-field intensity near to the receiver, consequently enhancing the efficiency of the WPT system [40]. Figure 4b shows the field when a defect cavity is constructed. The H-field strongly localizes at the cavity region. This strong localization of the H-field can further improve the WPT system’s performance.

**Figure 5:**
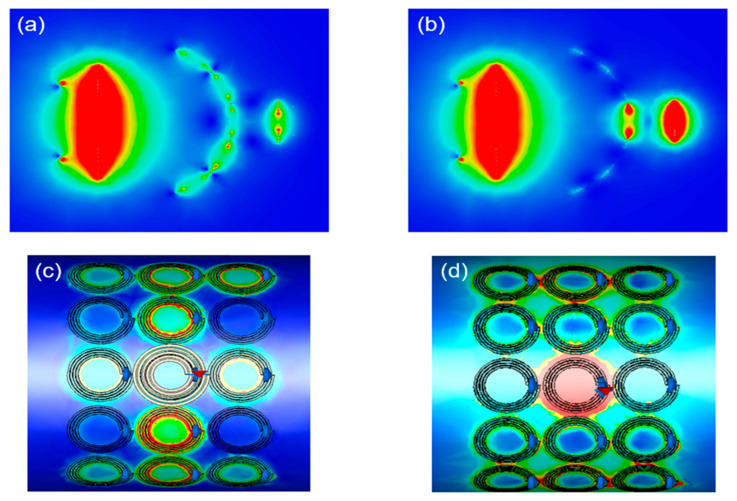
Field distributions in a WPT system with flexible metasurface (**a**) with a homogeneous metasurface and (**b**) with a non-homogeneous metasurface. Field distributions on metasurface (**c**) with a homogeneous metasurface and (**d**) with a non-homogeneous metasurface.

In several practical applications, a flat metasurface is unavailable or suboptimal. Therefore, the metasurface needs to be added to the WPT system as a bent shape. Figure 5a,b depict the H-field distributions in the WPT system corresponding to Figure 4a,b but with bent metasurfaces. When the metasurface is bent with a radius of 100 mm, surface waves still appear on the homogeneous metasurface, and the H-field is localized in the cavity of the non-homogeneous one. These results indicate that the metasurface can improve WPT’s efficiency under bending conditions. The H-field distributions on the metasurface for homogeneous and non-homogeneous metasurfaces are investigated in Figure 5c,d. A relatively uniform field distribution is observed in the homogeneous case, as shown in Figure 5c. The H-field is slightly higher at the center cell line of the metasurface, which can be attributed to the incident field of the Tx coil. In contrast, the field is significantly localized at the center cell of the metasurface in the non-homogeneous case, as shown in Figure 5d. This result confirms that cavity properties are maintained in the bending conditions. Note that, since the WPT system with circular shape Tx and Rx resonators work in the near-field regime, the metasurface can operate independently from the polarization of the magnetic field from the Tx resonator.

## 3. Experiment Results

**Figure 6:**
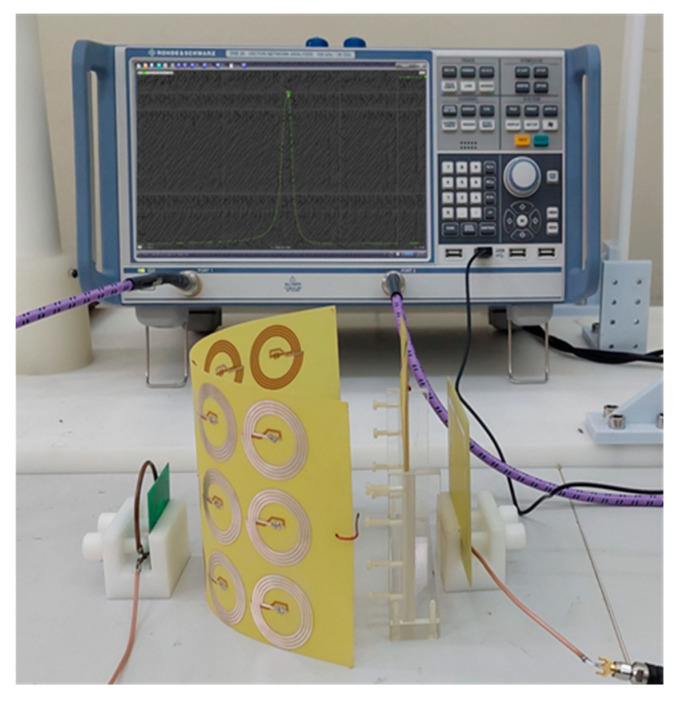
Experiment setup for the proposed WPT system with flexible metasurface.

A WPT system with a flexible metasurface was implemented to confirm the analysis in previous sections, as shown in Figure 6. The configuration of the system is similar to the simulation part in Figure 1. The Tx and Rx coils and the metasurface were fabricated by PCB technique on an FR-4 substrate with a thickness of 0.2 mm, thus minimizing fabrication errors and easily replicated. Thanks to such a thin thickness, the metasurface has the ability to bend in the shape of circles with a radius of curvature greater than 80 mm. The resonant frequency of Tx, Rx and unit cells on a metasurface is 13.56 MHz. The measured unloaded quality factors (*Q*-factors) of Tx, Rx, and metamaterial unit cells are 433, 165, and 152, respectively. The cavity on the metasurface can be formed by controlling the soldered capacitor values: 155 pF for the cavity and 180 pF for other cells in the metasurface. The WPT system’s characteristic was measured using a vector network analyzer (VNA-Rohde & Schwarz ZNB20, Rohde & Schwarz USA, Inc., Columbia, MD, USA). with a standard two-port calibration technique. After removing the impedance mismatch by adjusting the distance from Tx to the source loop (Rx to the load loop), both *S*_11_ and *S*_22_ are less than −10 dB. Therefore the transfer efficiency of the WPT system can be estimated using |*S*_21_|^2^ [29].

**Figure 7:**
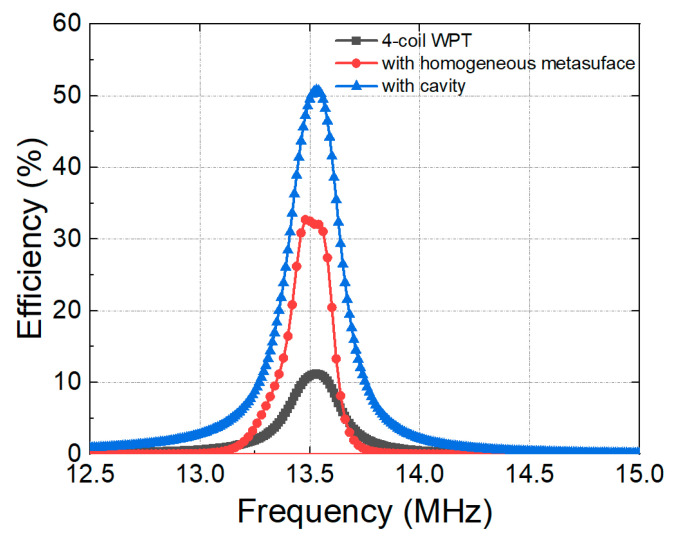
Measured efficiencies of WPT system with and without metasurface.

Figure 7 compares the measured efficiencies of WPT systems in three different configurations; original 4-coil WPT, WPT with a homogeneous metasurface, and WPT with a non-homogeneous metasurface. The distance between Tx and Rx was 200 mm, and the metasurface was positioned with *d*_Tx-Meta_ = 150 mm and *d*_Meta-Rx_ = 50 mm. The efficiency of the original 4-coil WPT achieved a maximum efficiency of 11.5% at 13.56 MHz, as depicted in the black curve. The efficiency is low because the distance between Tx and Rx in this configuration is 200 mm, which is four times greater than the diameter of Rx, thereby limiting the magnetic flux collected by Rx. The red curve shows the measured efficiency of the WPT system with a homogeneous metasurface. The peak efficiency obtained in this configuration was 32.3%, which is three times greater than that of the original system. Due to the mutual coupling between unit cells on the metasurface, the peak of efficiency is slightly expanded compared with the original case [28]. When a cavity is created in the metasurface, the peak efficiency enhances significantly to 52%, as shown by the blue curve. This is consistent with the simulation results (Figure 4) since the magnetic field is strongly localized in the cavity region.

**Figure 8:**
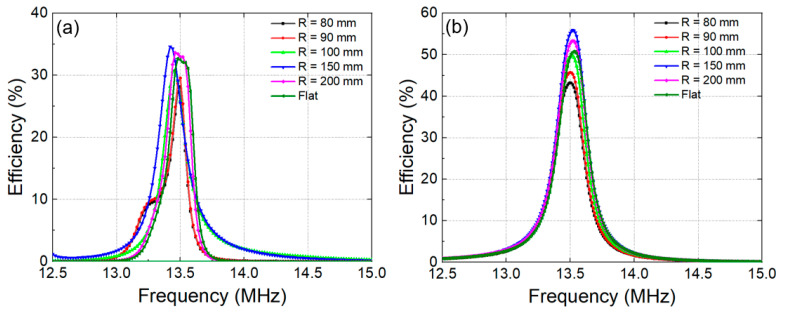
Measured efficiencies of WPT system as a function of frequency with various bending radii of (**a**) homogeneous metasurface and (**b**) non-homogeneous metasurface.

The performance of the WPT system with a flexible metasurface was investigated, as shown in Figure 8. Figure 8a presents the results of the WPT system with a homogeneous metasurface at various bending radii decreasing from 200 to 80 mm. At the minor curvatures corresponding to large bending radii, the efficiency of the WPT increased slightly compared to the flat configuration. This can be explained because a bending metasurface can collect more magnetic flux due to the closer distance between unit cells in the metasurface with Tx than in the flat case. The efficiency of WPT increased to 33.1% when the bending radius was 200 mm and reached 34.8% when the bending radius was 150 mm. When the curvature increased, corresponding to smaller bending radii, the efficiency decreased compared to the flat configuration. The efficiencies obtained were 31, 30 and 28.5%, with bending radii of 100, 90 and 80 mm, respectively. It can be noted that performance is reduced at small bending radii in these cases because the metasurface is bent too strongly. This might lead to the effective area being excessively reduced, thus limiting the collected magnetic flux. Figure 8b shows the measured efficiencies of the WPT system with a bending non-homogeneous metasurface. In this configuration, the system performance also increases at large bending radii and decreases at small bending radii. The maximum efficiency that can be achieved at a 150 mm radius of bending is 55.1%. At the smallest bending radius of 80 mm, the efficiency degraded to 42.5%. The WPT system with metasurface in various bending radii still shows significant efficiency enhancement compared with the original system.

In summary, with the flexible metasurface, we can obtain a maximum efficiency of 55.1% at the transfer distance larger than four times Rx diameter. At the same transfer distance and larger receiver, the previous work in [31] obtained an efficiency of 20% with reconfigurable metamaterial. In [37], the achieved efficiency of the WPT system with metasurface for a receiver was 50.4% at a distance of 120 mm.

## 4. Conclusions

In this work, we exploited the flexible metasurface state to improve the efficiency of the WPT system. Both homogeneous and non-homogeneous metasurfaces with a defect cavity were investigated at various bending radii. The field distributions of metasurfaces observed by EM simulation show that the magnetic field can be localized at the cavity region in the metasurface, thereby improving the WPT efficiency. The experiment setup with various bending radii was carried out to investigate the effect of a flexible metasurface on WPT performance. The WPT efficiency increases at the large bending radius, whereas a performance decrease was observed at the small bending radius. The maximum efficiency achieved in the presence of a flexible non-homogeneous metasurface is 55.1% compared to 32.3% and 11.5% of flat homogeneous metasurface and original WPT systems, respectively. The flexible metasurface can be more easily integrated into WPT systems than a flat metasurface. The flexible metasurface also can be further miniaturized for use in wearable devices. The results on the effectiveness of the flexible metasurface can expand the application range of metamaterial-based WPT systems, such as asymmetric WPT and non-coplanar WPT.

## Data Availability

The data presented in this paper are available on request from the corresponding author.
